# Short-term metabolic adjustments in *Plasmodium falciparum* counter hypoxanthine deprivation at the expense of long-term viability

**DOI:** 10.1186/s12936-019-2720-3

**Published:** 2019-03-19

**Authors:** Shivendra G. Tewari, Krithika Rajaram, Patric Schyman, Russell Swift, Jaques Reifman, Sean T. Prigge, Anders Wallqvist

**Affiliations:** 1 The Henry M. Jackson Foundation for the Advancement of Military Medicine, Inc. (HJF), Bethesda, MD USA; 2 Department of Defense Biotechnology High Performance Computing Software Applications Institute, Telemedicine and Advanced Technology Research Center, U.S. Army Medical Research and Materiel Command, Ft. Detrick, MD USA; 3 Department of Molecular Microbiology and Immunology, Johns Hopkins University, Baltimore, MD USA

**Keywords:** Purine deprivation, *Plasmodium falciparum*, Transcriptome, Metabolome, Metabolic network model, Gene set enrichment analysis, Stress response pathways

## Abstract

**Background:**

The malarial parasite *Plasmodium falciparum* is an auxotroph for purines, which are required for nucleic acid synthesis during the intra-erythrocytic developmental cycle (IDC) of the parasite. The capabilities of the parasite and extent to which it can use compensatory mechanisms to adapt to purine deprivation were studied by examining changes in its metabolism under sub-optimal concentrations of hypoxanthine, the primary precursor utilized by the parasite for purine-based nucleic acid synthesis.

**Methods:**

The concentration of hypoxanthine that caused a moderate growth defect over the course of one IDC was determined. At this concentration of hypoxanthine (0.5 μM), transcriptomic and metabolomic data were collected during one IDC at multiple time points. These data were integrated with a metabolic network model of the parasite embedded in a red blood cell (RBC) to interpret the metabolic adaptation of *P. falciparum* to hypoxanthine deprivation.

**Results:**

At a hypoxanthine concentration of 0.5 μM, vacuole-like structures in the cytosol of many *P. falciparum* parasites were observed after the 24-h midpoint of the IDC. Parasites grown under these conditions experienced a slowdown in the progression of the IDC. After 72 h of deprivation, the parasite growth could not be recovered despite supplementation with 90 µM hypoxanthine. Simulations of *P. falciparum* metabolism suggested that alterations in ubiquinone, isoprenoid, shikimate, and mitochondrial metabolism occurred before the appearance of these vacuole-like structures. Alterations were found in metabolic reactions associated with fatty acid synthesis, the pentose phosphate pathway, methionine metabolism, and coenzyme A synthesis in the latter half of the IDC. Furthermore, gene set enrichment analysis revealed that *P. falciparum* activated genes associated with rosette formation, Maurer’s cleft and protein export under two different nutrient-deprivation conditions (hypoxanthine and isoleucine).

**Conclusions:**

The metabolic network analysis presented here suggests that *P. falciparum* invokes specific purine-recycling pathways to compensate for hypoxanthine deprivation and maintains a hypoxanthine pool for purine-based nucleic acid synthesis. However, this compensatory mechanism is not sufficient to maintain long-term viability of the parasite. Although *P. falciparum* can complete a full IDC in low hypoxanthine conditions, subsequent cycles are disrupted.

**Electronic supplementary material:**

The online version of this article (10.1186/s12936-019-2720-3) contains supplementary material, which is available to authorized users.

## Background

In 2016, there were 216 million cases of malaria and 445,000 malaria-related deaths worldwide [1], with 90% of these incidents occurring in Africa. *Plasmodium falciparum*, the most lethal malaria parasite, is responsible for 99% of malaria-related deaths in sub-Saharan Africa [1]. During the symptomatic stage of the disease, i.e., during the blood stage, a single merozoite reproduces asexually within a human red blood cell (RBC) to form 16–32 merozoites [2, 3], which go on to invade other RBCs to begin another cycle. During one such cycle, the original merozoite needs to synthesize several macromolecules, such as DNA, RNA and lipids, to successfully reproduce and complete its intra-erythrocytic developmental cycle (IDC).

**Fig. 1:**
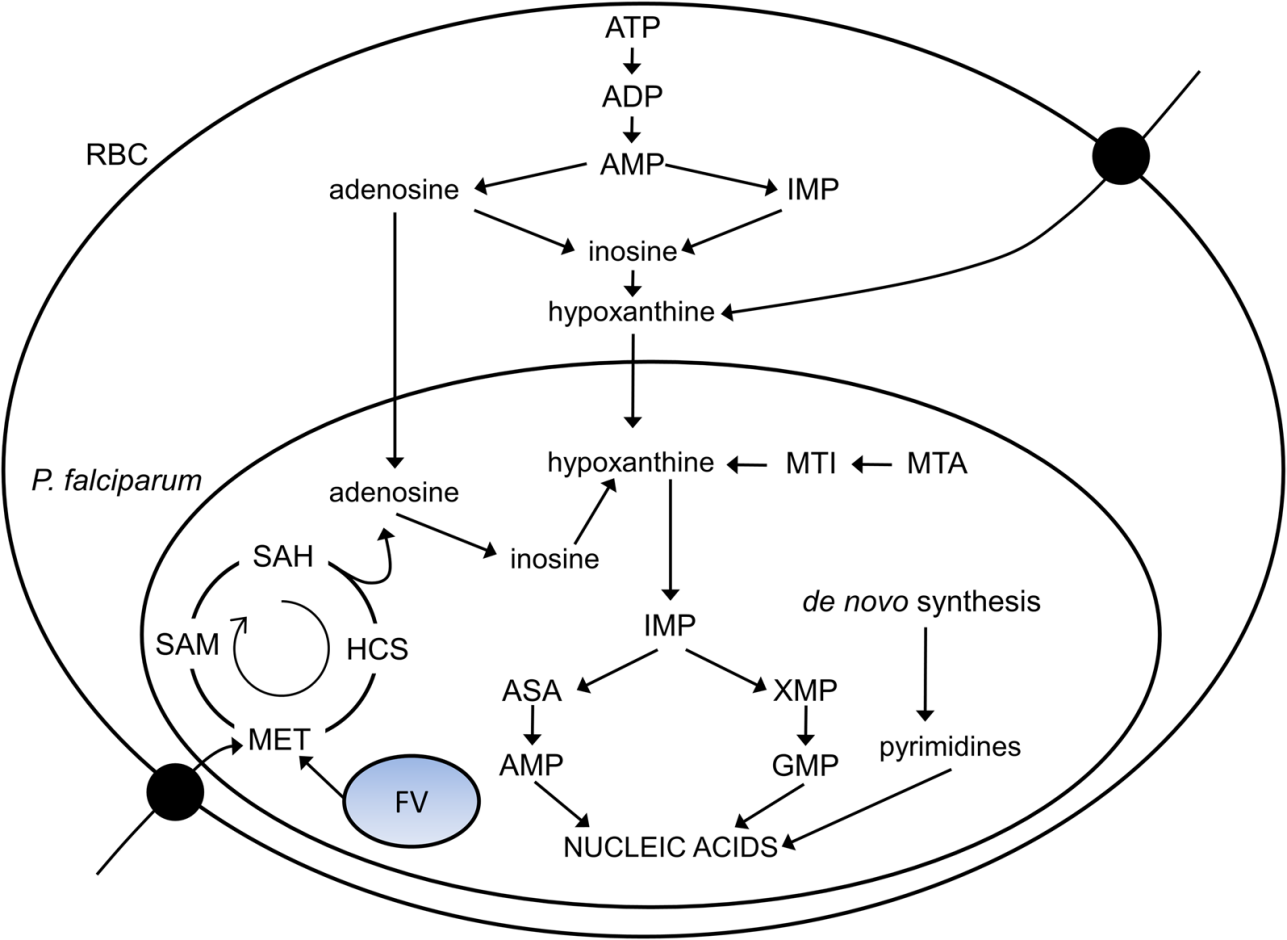
Schematic showing hypoxanthine salvage in *Plasmodium falciparum.* Hypoxanthine in the cytosol of a red blood cell (RBC) can be formed by catabolism of adenosine triphosphate (ATP) or imported through hypoxanthine transporters from the environment, where it is present at 2–8 µM in human serum. The parasite takes up hypoxanthine from the RBC cytosol and uses it to make purine-based nucleic acids, whereas it synthesizes pyrimidine-based nucleic acids de novo. The parasite can produce hypoxanthine from methylthioinosine (MTI) and methylthioadenosine (MTA), which are produced during polyamine synthesis. The parasite can also produce hypoxanthine from adenosine, which it either takes up from the RBC’s cytosol or by directly cleaving *S*-adenosylhomocysteine (SAH). SAH is synthesized from methionine (MET), which the parasite acquires through hemoglobin digestion in the food vacuole (FV) or via plasma membrane transporters. Black dots on the RBC membrane indicate hypoxanthine and MET transporters. ADP, adenosine diphosphate; AMP, adenosine monophosphate; ATP, adenosine triphosphate; ASA, adenylosuccinic acid; GMP, guanine monophosphate; HCS, homocysteine; IMP, inosine monophosphate; SAM, *S*-adenosylmethionine; XMP, xanthine monophosphate

In contrast to most human cells, *P. falciparum* parasites [4] and RBCs [5] lack the machinery to synthesize purine bases de novo. The RBC purine pool is mostly adenosine triphosphate (ATP) [6], but that is not sufficient to support continuous parasite growth during the IDC under in vitro conditions [4]. Therefore, during the IDC, the parasite needs an extra-erythrocytic supply of purines to maintain successful nucleic acid synthesis. Considerable evidence indicates that RBC hypoxanthine is the primary precursor utilized by the parasite for purine-based nucleic acid synthesis under in vivo conditions [6–8]. Figure 1 shows a schematic of the hypoxanthine salvage process in a parasite-infected RBC [4, 5]. Previous experiments have shown that depletion of RBC hypoxanthine inhibits parasite growth under in vitro growth conditions [5, 6].

To determine how hypoxanthine deprivation affects parasite metabolism, *P. falciparum* was first cultured under a range of hypoxanthine concentrations to identify appropriate nutrient-deprived conditions. At a concentration of 0.5 μM, transcriptomic and metabolomic data were first collected and then integrated with metabolic network models of uninfected and parasite-infected RBCs to predict the metabolism of *P. falciparum* under hypoxanthine-deprivation conditions. Model simulations of uninfected and parasite-infected RBCs were validated by comparing them with relevant experimental data. Lastly, the metabolic adaptations of the parasite under hypoxanthine-deprivation conditions were investigated and gene set enrichment analysis (GSEA) was performed to identify biological processes that were altered significantly beyond metabolic adaptations. The GSEA results were used to identify pathways and processes invoked by the parasite in response to hypoxanthine deprivation. These pathways and processes were then contrasted with those invoked in response to a different nutrient-deprivation condition [9], to identify common and distinct aspects of the two nutrient stress conditions.

## Methods

### Parasite culture, hypoxanthine deprivation, and sample generation

*Plasmodium falciparum* Nf54^attB^ parasites (a gift from David Fidock, Columbia University) were cultivated in RBCs at 2% haematocrit (HCT) under a mixed gas atmosphere (94% N_2_, 3% O_2_, and 3% CO_2_) at 37 °C. The parasite cultures were maintained in Roswell Park Memorial Institute (RPMI)-1640 medium (Gibco, Gaithersburg, MD, USA) supplemented with 25 mM HEPES, 90 µM hypoxanthine, 0.3% sodium bicarbonate, 25 µg/mL gentamicin, 0.5 µM *R*-lipoic acid, and 0.5% AlbuMAX II (Life Technologies, Carlsbad, CA, USA). This medium is referred to as the hypoxanthine-rich medium.

To determine the hypoxanthine-deprivation conditions, experiments were first carried out in 24-well culture plates to identify the concentration range in which parasite viability was affected. RBCs containing late-stage parasites were collected by Magnetic Activated Cell Sorting (MACS). Parasite-infected RBCs were purified and diluted in fresh hypoxanthine-rich medium to approximately 0.8% parasitaemia with 2% HCT. The culture was centrifuged (1600×*g*, 5 min) to pellet the RBCs within 0–2 h after they were infected. The pellet was washed once in medium lacking hypoxanthine and then seeded into a medium containing a hypoxanthine concentration of 0, 0.5, 2, 5, or 90 µM. Parasite morphology and parasitaemia were monitored by Giemsa staining of blood smears at 24 and 40 h. To assess re-infection of RBCs, a tenth of each culture was transferred into fresh hypoxanthine-rich medium with 2% HCT at 40 h. Blood smears were prepared at 72 h for these diluted cultures to assess re-infection of RBCs.

Parasite samples for transcriptomic and metabolomic analyses were prepared according to the methods described by Tewari et al. [10]. Briefly, for each IDC, synchronous parasite cultures were maintained by passing them through cell-sorting MACS columns for a period of 2 weeks, while scaling the culture volume up to 300 mL. After the final passage through a cell-sorting MACS column, the cultures were divided into four 75-mL volumes at 2% HCT using Percoll-purified RBCs. The cultures were pelleted and washed once in hypoxanthine-free medium within 0–2 h after they were infected by the parasite. The pellets were washed and re-suspended in 300 mL of hypoxanthine-deprived medium. They were divided again into four 75-mL cultures, and this time point was denoted as 0 h. Quadruplicate uninfected RBC cultures were also prepared in four 50-mL volumes to provide control samples for metabolomic analyses. Samples were collected at seven time points during the IDC (at 0, 8, 16, 24, 36, 40, and 48 h). The quadruplicate samples were sent to the Johns Hopkins Genomic Analysis and Sequencing Core Facility for transcriptomic analysis using Agilent microarray chip AMADID 037237 (Agilent Technologies, Inc., Santa Clara, CA, USA) and to Metabolon, Inc. (Durham, NC, USA) for metabolomic analysis.

### Viability of the parasites under continued deprivation

To determine how long hypoxanthine-deprived parasites remain viable, parasites were cultured using the methods described above at hypoxanthine concentrations of 90 µM (hypoxanthine-rich) and 0.5 (hypoxanthine-deprived) for over a period of 120 h, and blood smears were prepared at 24, 40, and 48 h. A tenth of the hypoxanthine-deprived culture was transferred to hypoxanthine-rich medium at 40, 48, 60, 72, and 96 h, while the hypoxanthine-rich culture was transferred at 40 h to serve as a control. Twenty-4 h after transfer, the cultures were assessed for parasitaemia using a blood smear test. All growth experiments were carried out in triplicate wells for each condition and the procedure was repeated at least twice.

### Metabolic network model and data processing

The present study used the latest version of a *P. falciparum* metabolic network model, which consists of 1025 metabolic reactions and 923 metabolites [10, 11]. This model has been annotated to include the PubChem IDs [12] of 273 metabolites found in metabolomic data collected from the *P. falciparum* Nf54^attB^ strain maintained in RPMI-1640 medium during the IDC, as determined by (1) ultrahigh-performance liquid chromatography (UPLC) tandem mass spectrometry (MS/MS) [10]; and, (2) metabolomic data obtained under isoleucine deprivation [9].

**Fig. 2:**
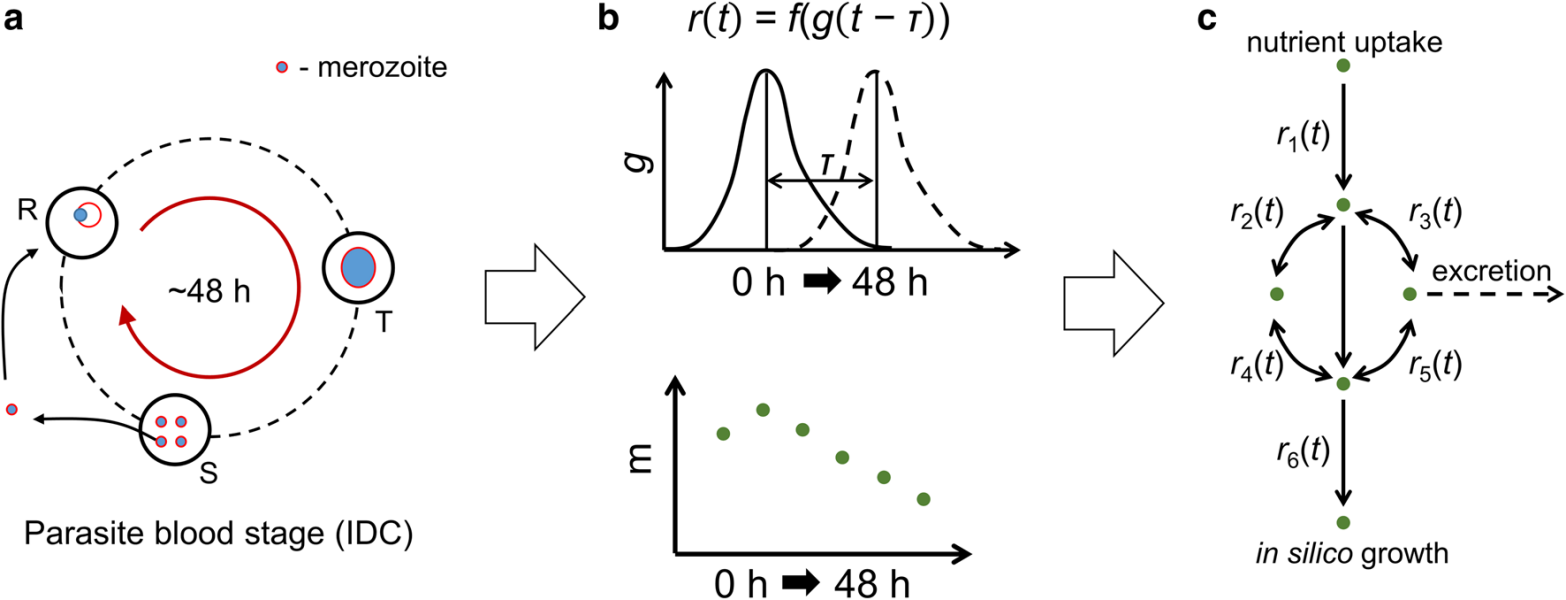
Schematic diagrams showing integration of transcriptomic and metabolomic data with the metabolic network model. **a** The typical life cycle of *P. falciparum* during the blood stage, also known as the intraerythrocytic developmental cycle (IDC). The cycle begins when a merozoite infects a red blood cell (RBC: circle drawn with a solid black line). Subsequently, the parasite develops and multiplies within the RBC to form rings (R), trophozoites (T), and schizonts (S). Once the parasite completes the cycle, it ruptures the RBC to release 16–32 merozoites [2, 3], which in turn invade another RBC to begin another cycle. The typical life cycle of *P. falciparum* lasts about 48 h. **b** Upper panel: representation of gene transcription during the 48-h IDC. There is typically a time delay (denoted by τ) between the transcription of a gene and the translation of an enzyme. In the computational framework of the present study, this delay is reflected in the shift of *r*(*t*) relative to the function for gene transcription [denoted by *f*(*g*)]. Lower panel: abundance of a metabolite (m) during the 48-h IDC. In the present framework, the relative abundance of a participant metabolite is assumed to modulate the flux through a given metabolic reaction. **c** A metabolic network model showing integration of metabolic and transcriptomic information. In this panel, *r*_1_(*t*) alters nutrient uptake of the model and affects ‘in silico growth’ *r*_6_(*t*), given other metabolic reactions [*r*_2_(*t*) to *r*_5_(*t*)] and secretion processes in the model

The gene-to-reaction mapping of the metabolic network model was used to compute *r*, the level of expression of an enzymatic reaction, which is a function of the gene(s) transcribing the enzyme(s) catalyzing a given reaction (Fig. 2b). Specifically, for a metabolic enzyme encoded by a single gene, *r* was assumed to be identical to the transcription of that gene. To compute *r* for such a metabolic enzyme at a given time point in the IDC, the expression level of the encoding gene at time *t*–τ was used (where ‘τ’ denotes the transcriptional delay, as described in Foth et al. [13]). For a metabolic enzyme encoded by more than one gene, Boolean rules (AND/OR gate) were used to compute *r*. These rules were implemented by taking the maximum expression value of the genes encoding an enzyme if they were connected by an ‘AND’ gate, or the minimum expression value if they were connected by an ‘OR’ gate [14].

### Estimating alterations in parasite-infected RBC metabolism in response to hypoxanthine deprivation

Figure 1 shows that an RBC can synthesize hypoxanthine from inosine via purine nucleoside phosphorylase (PNP). However, in practice, the free energy of the PNP reaction favours the formation of inosine from hypoxanthine. Therefore, any alteration in RBC hypoxanthine levels could alter metabolic reactions within the RBC as well. The present study used a recently developed method [10] that predicts metabolic reaction fluxes from metabolomic data. This method was used to identify alterations in parasite-infected RBC metabolism in response to hypoxanthine deprivation. The optimization steps used to predict the metabolism of parasite-infected RBC under hypoxanthine-rich and -deprived conditions are provided in Additional file 1: Text S1.

### Simulating the effect of hypoxanthine deprivation on *Plasmodium falciparum* metabolism

A previously described computational framework was used for simulating hypoxanthine deprivation [15]. Briefly, alterations in transcriptomic data obtained under control and stressed conditions were used to simulate and describe the stress phenotype, and the approach was modified to include the effects of metabolite pool alterations, which may occur during the IDC under different conditions. Specifically, time-dependent metabolomic data obtained under hypoxanthine-rich and -deprived conditions were used as an additional constraints to the previous approach [15].

To obtain the metabolic flux distribution of *P. falciparum* during the IDC, the following equations were solved:

1 $${\text{Minimize}}\sum\limits_{j \in R} {\left| {v_{j} } \right|}$$

$${\text{subject to:}} \, S \cdot \overline{v} = 0$$

$$\sum\limits_{i \in M} {\left| {v_{i} - \widetilde{{m_{i} }} \cdot v_{i,nom} } \right|} < \delta$$

$$v_{g} = \mu$$

where *R* denotes the set of all metabolic reactions of *P. falciparum*, *M* represents the set of metabolic reactions influenced by the metabolomic data, *S* represents a matrix containing the stoichiometry coefficients of all reactions, $\overline{v}$ denotes a column vector containing all metabolic reactions of the network, $\delta$ denotes the minimum of $\mathop \sum _{i \in M} \left| {v_{i} - \widetilde{{m_{i} }} \cdot v_{i,nom} } \right|$ obtained separately, *v*_g_ represents the metabolic reaction governing the growth of the parasite, $\mu$ denotes the nominal value of the parasite growth rate, which is set to 0.48 g/h gDW of the original merozoite [2], and $\widetilde{{m_{i} }}$ denotes the minimum value of $m_{i}$, where $m_{i}$ is a vector containing the median values of each metabolite taking part in the *i*th metabolic reaction. $v_{i,nom}$ denotes the *i*th value of $v_{nom}$, which is obtained by solving the following:

2 $$\hbox{min} \sum\limits_{j \in R} {\left| {v_{j} } \right|}$$

$${\text{subject to:}} \, v_{i} < v_{N} ,\quad \forall \, i \in N$$

$$v_{g} = \mu$$

here, *N* denotes the set of reactions transporting nutrients across the parasite plasma membrane and $v_{N}$ denotes a vector containing the optimal values of every reaction in *N*. The other variables are as defined above.

The optimization problems shown in Eqs. (1) and (2) yield *v*_ref_, which were modulated using the time-dependent transcriptomic and metabolomic data to obtain the temporal profile of *P. falciparum* metabolism. The time-dependent transcriptomic and metabolomic data were incorporated by solving the following:

3 $$\hbox{min} \sum\limits_{j \in G;j \ne i} {\left| {v_{j}^{t} - r_{j}^{t} v_{j,ref} } \right|} + \sum\limits_{i \in G} {\left| {v_{i}^{t} - \overline{m}_{i}^{t} \cdot v_{i,ref} } \right|}$$

$${\text{subject to:}} \,S \cdot \overline{v}^{t} = 0$$

$$\sum\limits_{i \in G} {\left| {v_{i}^{t} - \overline{m}_{i}^{t} \cdot v_{i,ref} } \right|} \le \varepsilon$$

$$v_{k} < v_{N} ,\quad \forall \, k \in N.$$

here, *G* denotes the set of all intracellular reactions of *P. falciparum*, ${\text{r}}_{\text{j}}^{\text{t}}$ denotes the reaction expression of the *j*th reaction at time *t*, $\overline{m}_{i}^{t}$ represents the influence of a metabolite at time *t* for the *i*th reaction, and the bar above *m* denotes normalization of metabolite abundance by its median over the IDC. $v_{j,ref}$ and $v_{i,ref}$ represent the *j*th and *i*th values, respectively, of $v_{ref}$. As suggested by Fang et al. [2], the problem shown in Eq. (3) can have multiple solutions. Therefore, the following optimization problem was solved to obtain a solution closest to $v_{ref}$:

4 $$\hbox{min} \, \left\| {v^{t} - v_{ref} } \right\|$$

$${\text{subject to:}}\sum\limits_{j \in G;j \ne i} {\left| {v_{j}^{t} - r_{j}^{t} v_{j,ref} } \right|} + \sum\limits_{i \in G} {\left| {v_{i}^{t} - m_{i}^{t} \cdot v_{i,ref} } \right|} \le \delta$$

$$v_{i} < v_{N} ,\quad \forall \, i \in N$$

The method to solve the optimization problems in Eqs. (2)–(4) was identical to the method previously described [15], except for the term incorporating the metabolomic data in Eq. (3).

### Gene set enrichment analysis (GSEA)

GSEA was performed based on the method used in a previous study [16]. The absolute value of each gene’s log-transformed fold-change value was first calculated under control and stress conditions. The maximum absolute fold-change value of DNA probes that mapped to one gene were used. Next, the mean (*μ*) and standard deviation (*σ*) of the absolute values were calculated based on the absolute fold-change values of all genes. The average score of a given gene set ($\overline{X}$) was computed by taking the mean of the absolute fold-change values associated with a given gene set. The enrichment of a gene set was estimated by its *p* value, i.e., the probability of observing a score ($\overline{X}$) by chance. According to the Central Limit Theorem, the probability distribution of an average value is approximately normal with parameters *μ* and $\sigma /\sqrt n$, where *n* is the number of genes in a given gene set. The *p*-value can be calculated from the z-transform of the average score of a given gene set, i.e., $z = \frac{{\overline{X} - \mu }}{\sigma /\sqrt n }$. GSEA was performed using the gene ontology information for *P. falciparum*, which is available through the GO2MSIG platform [17].

## Results

### Effect of hypoxanthine deprivation on *Plasmodium falciparum* during the IDC

**Fig. 3:**
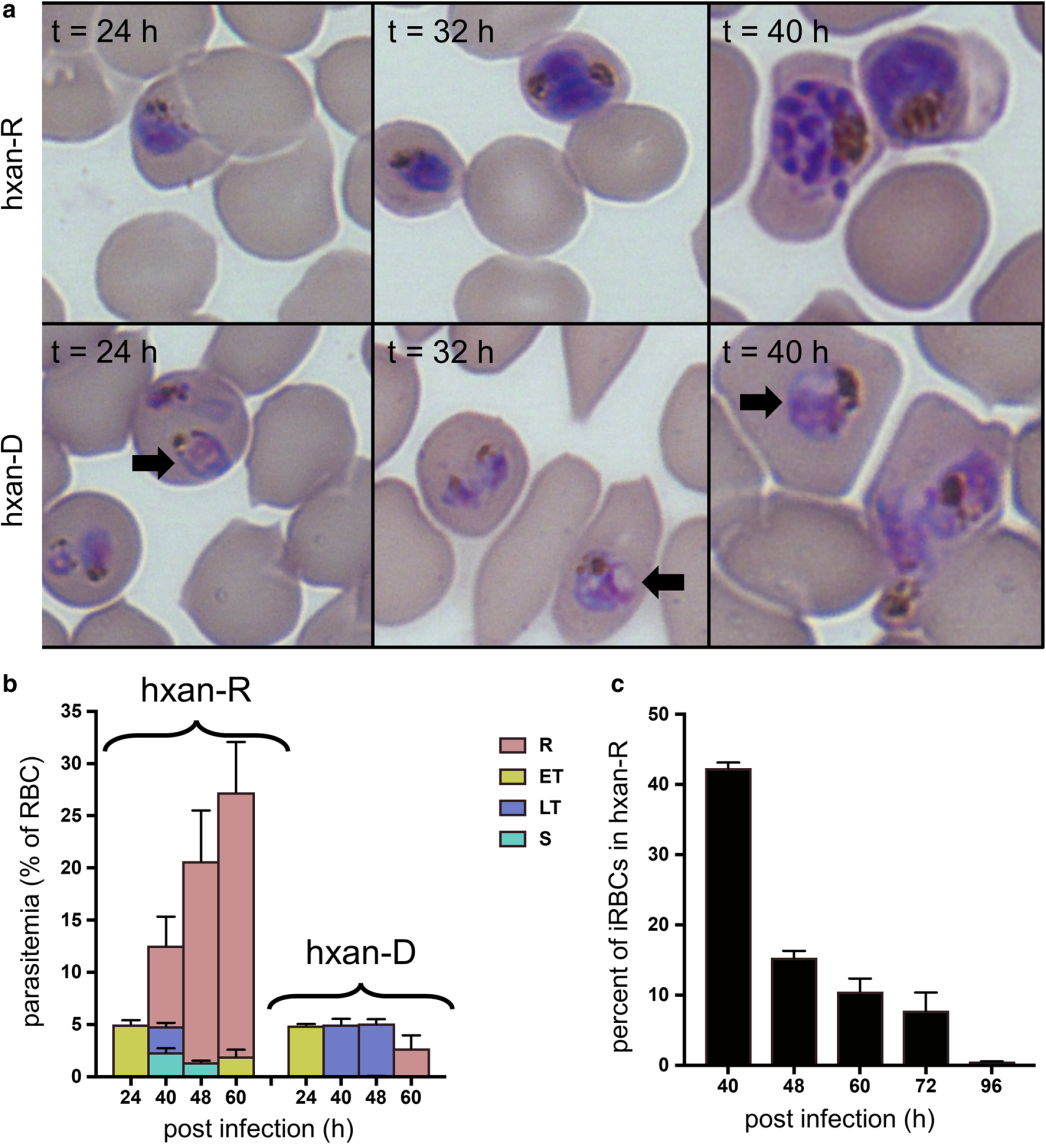
Effect of hypoxanthine deprivation on *Plasmodium falciparum* during the IDC. **a** Representative images of Giemsa-stained parasites at 24, 32 and 40 h in hypoxanthine-rich medium (hxan-R) and hypoxanthine-deprived medium (hxan-D). Black arrows indicate vacuole-like structures in the cytoplasm of the parasite. **b** Developmental-stage specific parasitaemia under the two culture conditions (hxan-R and hxan-D) at different time points. **c** Recovered parasite numbers shown as a percentage of parasite-infected RBCs (iRBCs) maintained in hypoxanthine-rich medium at 40 h into the IDC. The number of recovered parasites is calculated 24 h after the transfer of the deprived parasites to the rich medium. ET, early trophozoite; hxan-D, hypoxanthine-deprived; hxan-R, hypoxanthine-rich; LT, late trophozoite; R, ring; S, schizont

*Plasmodium falciparum* parasites are normally cultured in the presence of 90–360 μM added hypoxanthine. Previous studies have shown that hypoxanthine concentrations as low as 2 μM can support *P. falciparum* IDC completion [18], but at least 5 μM is required for optimal growth. To determine how parasites respond to lower levels of hypoxanthine, synchronized parasites were cultured for 40 h at different hypoxanthine concentrations, after which they were transferred to a hypoxanthine-rich medium (90 μM hypoxanthine). The parasites cultured in 0.5 µM hypoxanthine during the first 40 h experienced approximately a 50% reduction in parasitaemia (assessed at 72 h) when compared with the parasites cultured in hypoxanthine-rich medium (Additional file 1: Fig. S1). This concentration was selected to further study the effect of hypoxanthine deprivation on the parasite development. The parasite numbers and morphology in hypoxanthine-rich and -deprived conditions were comparable for the first 24 h, after which vacuole-like structures became visible in the cytosol of hypoxanthine-deprived parasites. Figure 3a (lower panel) shows these vacuole-like structures (black arrows).

The parasites in the rich medium underwent schizogony between 40 and 48 h. By contrast, the deprived parasites were in the trophozoite stage at 40 h and about 18% of the total parasites were schizonts at 48 h. There was no re-infection of RBCs during the 48-h IDC. Figure 3b shows that hypoxanthine-deprived parasites only developed rings at 60 h. To determine how long these deprived parasites remain viable, they were transferred to a hypoxanthine-rich medium after various lengths of hypoxanthine deprivation (i.e., at 40, 48, 60, 72, and 96 h). Figure 3c shows that the parasites were able to establish a second IDC when they were transferred between 40 and 72 h, but not after 72 h.

### Effect of hypoxanthine deprivation on parasite-infected RBC metabolism

Malaria parasites require hypoxanthine to synthesize purine-based nucleotides. Both the parasite and the RBC have PNP enzymes, which can produce hypoxanthine from inosine. Because the parasite relies on RBC metabolism for a number of nutrients during the IDC, the data were first analysed to assess the impact of hypoxanthine deprivation on parasite-infected RBC metabolism per se.

Parasite-infected RBC metabolism did not significantly change in response to hypoxanthine deprivation. In fact, the metabolic pathways of a parasite-infected RBC were perturbed to a similar degree regardless of the culture medium. Table 1 lists selected metabolic pathways in the RBC and the number of reactions within each pathway that were significantly altered (*p* < 0.01) in response to parasite infection under hypoxanthine-rich and -deprived conditions. A complete list of the RBC metabolic pathways perturbed under these two conditions is provided in Additional file 2.

**Table 1:** Model-predicted changes in parasite-infected RBC metabolism

Metabolic pathway	Number of metabolic reactions	
	Hypoxanthine-rich medium (90 µM)	Hypoxanthine-deprived medium (0.5 µM)
Phospholipid	7	8
Haem synthesis	7	7
Pentose phosphate pathway	1	1
Haem degradation	3	3

Statistically significant reactions, between simulations of uninfected and parasite-infected erythrocyte metabolism, computed using Student’s *t*-test and a criterion of *p* < 0.01

### Effect of hypoxanthine deprivation on key purine metabolites

**Fig. 4:**
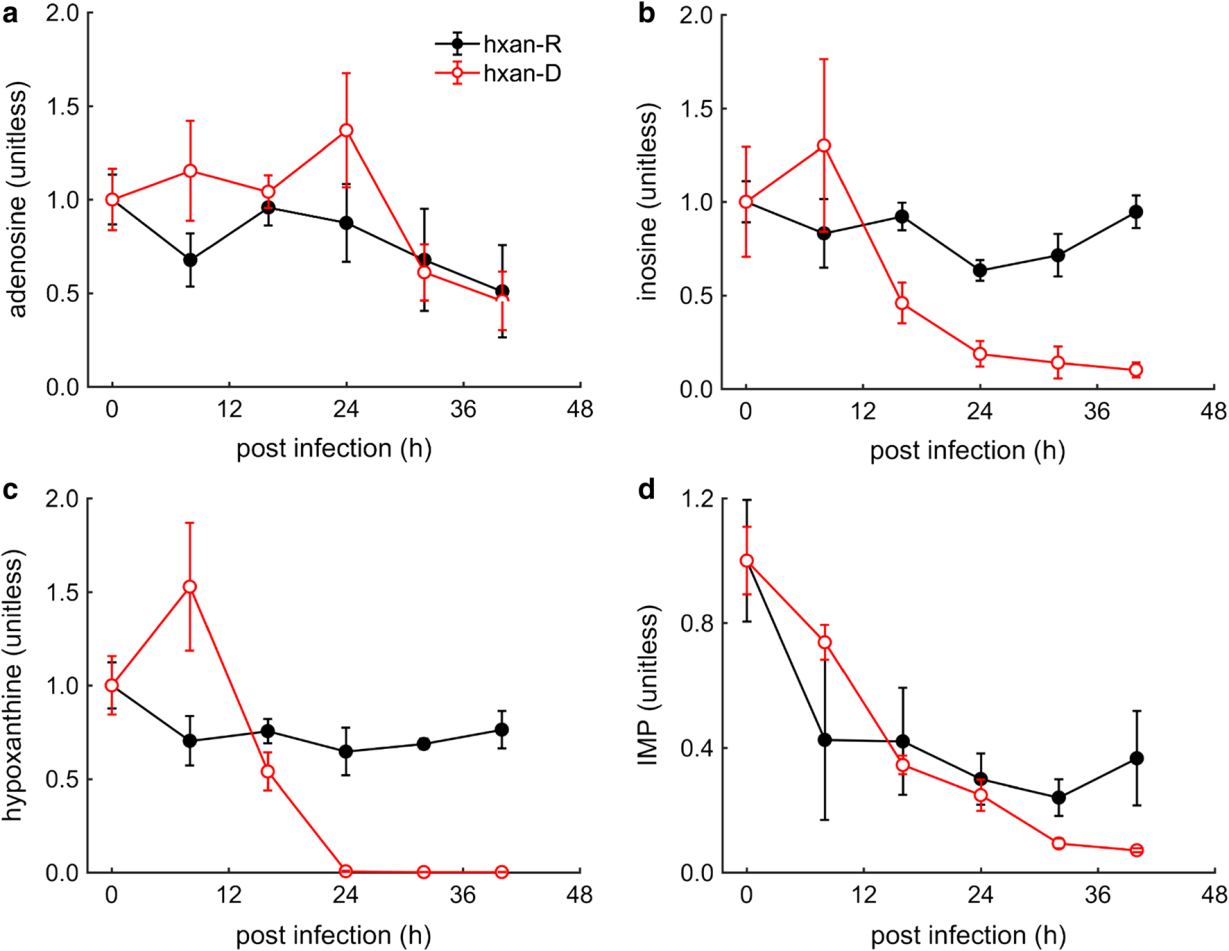
Key purine metabolites under hypoxanthine-rich (hxan-R) and -deprived (hxan-D) conditions. **a** Adenosine, **b** inosine, **c** hypoxanthine, and **d** inosine monophosphate (IMP) levels from parasite-infected red blood cells under the two conditions. At the first two time points of hypoxanthine deprivation, each metabolite was higher than its value under the hypoxanthine-rich condition. During the second day of infection under the hypoxanthine-deprived condition, inosine and hypoxanthine levels were substantially lower than their values under the hypoxanthine-rich condition. To allow comparison of a metabolite between the two conditions, the value of a given metabolite was normalized by its initial value at time 0 h. The error bars show the standard deviations of measurements from four technical replicates

In the culture conditions used here, malaria parasites had access to only hypoxanthine as their sole purine source, which was kept at a pre-determined low concentration of 0.5 µM. Figure 4 shows key purine metabolites that were used by the parasite to synthesize purine-based nucleotides, indicating that hypoxanthine levels under the deprivation condition only dropped after the 24-h time point. By contrast, Fig. 4a and d show that the levels of adenosine and inosine monophosphate (IMP) under the hypoxanthine-deprived condition were either higher than (or at least comparable to) those under the hypoxanthine-rich condition. Figure 4b depicts inosine levels, which resemble the pattern observed with hypoxanthine levels under deprivation. A comparison of Fig. 4c and d indicates that the parasite was able to maintain IMP levels during the second day of the 48-h IDC despite having relatively negligible quantities of hypoxanthine during this time period. A complete list of metabolites detected under hypoxanthine-rich and -deprived condition is provided in Additional file 3.

### Model simulations capturing the effect of hypoxanthine deprivation on parasite metabolism

**Fig. 5:**
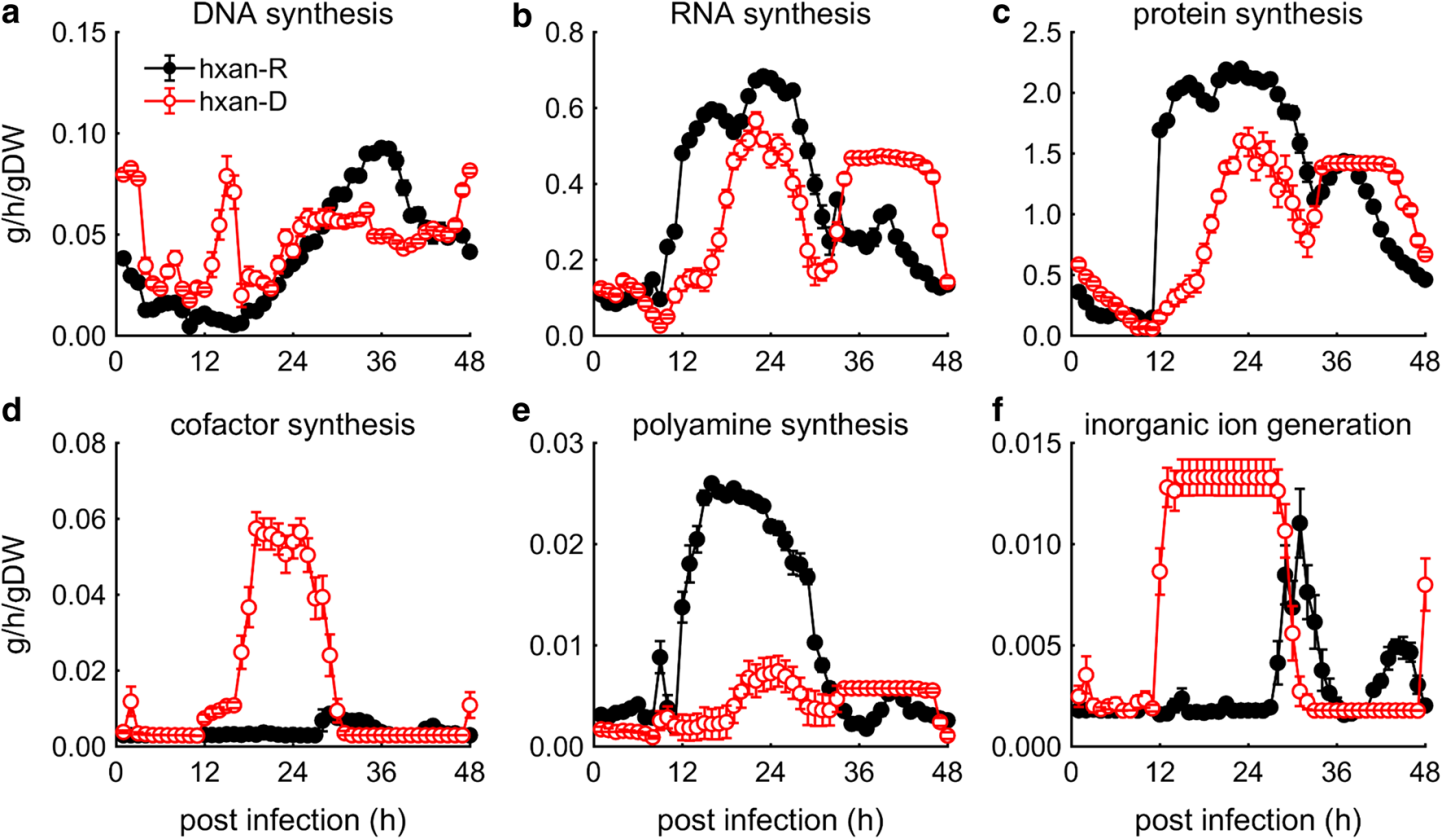
Model-predicted effect of hypoxanthine deprivation on major biomass components of *Plasmodium falciparum.* **a** DNA synthesis rate, **b** RNA synthesis rate, **c** protein synthesis rate, **d** cofactor synthesis rate, **e** polyamine synthesis rate, and **f** inorganic ion generation rate. The error bars show standard deviation of 50 simulations computed after adding random Gaussian noise with a mean of zero and a standard deviation of 5% to the transcriptomic data obtained under hypoxanthine-rich and hypoxanthine-deprived conditions. hxan-D, hypoxanthine-deprived; hxan-R, hypoxanthine-rich

Hypoxanthine is a key precursor of purine bases for *P. falciparum* to synthesize DNA, RNA, and protein. The extent to which the proposed model could capture the time-dependent changes in DNA, RNA, and protein synthesis over the course of the IDC in the hypoxanthine-deprived condition was examined (Fig. 5a–c). Compared to the hypoxanthine-rich condition (black plots), the hypoxanthine-deprivation simulations showed a slight increase in DNA synthesis during the first 24 h, followed by a slight decrease between 30 and 42 h, while both RNA synthesis and protein synthesis slightly decreased between 12 and 32 h. By contrast, hypoxanthine deprivation markedly altered the synthesis or generation of cofactors, polyamines, and inorganic ions relative to the hypoxanthine-rich condition (Fig. 5d–f).

As noted above, vacuole-like structures within the parasite cytoplasm became apparent at 24 h into the IDC (Fig. 3a). To identify the metabolic processes of *P. falciparum* that were significantly altered before and after the appearance of these structures under the hypoxanthine-deprived condition, model simulations of hypoxanthine-rich parasites and hypoxanthine-deprived parasites were compared to identify significantly altered metabolic reactions. Table 2 lists the top reactions and pathways of *P. falciparum* metabolism that were altered before and after the appearance of these structures. The additonal material lists the genes of each metabolic pathway perturbed within 24 h (Additional file 4, Sheet 3) or after 24 of infection (Additional file 4, Sheet 4).

**Table 2:** *Plasmodium falciparum* metabolic pathways altered in response to hypoxanthine deprivation

Within 24 h of infection		Past 24 h of infection	
Pathway	Number of reactions	Pathway	Number of reactions
Phospholipid metabolism	10	Fatty acid synthesis	34
Porphyrin metabolism	9	Pentose phosphate cycle	7
Ubiquinone metabolism	9	Methionine polyamine metabolism	6
Isoprenoid metabolism	8	CoA biosynthesis	5
Nicotinate metabolism	7	Lipid utilization	5
Shikimate biosynthesis	7	TCA cycle	5

Reactions that significantly differed between simulations of parasite metabolism under hypoxanthine-rich and -deprived conditions, as determined by Student’s *t*-test and a significance criterion of *p* < 0.01

CoA, coenzyme A; TCA, tricarboxylic acid

### Gene set enrichment analysis (GSEA) of stress response pathways

**Fig. 6:**
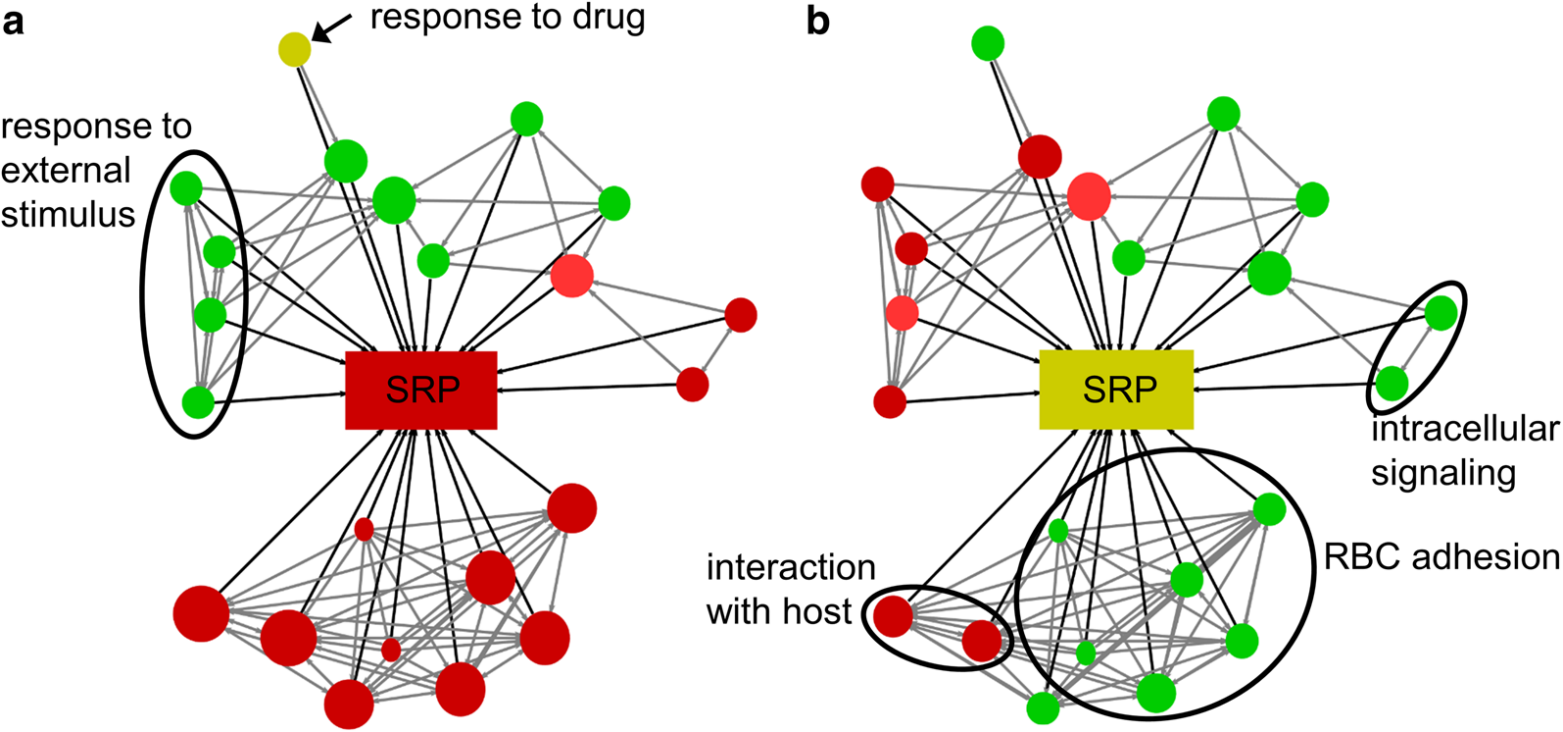
Gene set enrichment analysis of stress response pathways (SRPs) in *Plasmodium falciparum.* Results for transcriptomic data obtained under **a** hypoxanthine and **b** isoleucine deprivation. The figure shows gene ontologies associated with the SRPs of *P. falciparum.* Here each circle represents a gene ontology associated with the SRPs, where the size of an ontology circle is proportional to the number of genes in it. A black line drawn from a colored circle to the SRP indicates that this gene set directly influences the SRP. Similar gene sets are clustered together and grey lines are used to indicate interaction between them. The cluster named ‘interaction with host’ contains proteins associated with Maurer’s cleft and parasitic protein export. ‘RBC adhesion’ contains *P. falciparum* red blood cell (RBC) membrane protein 1 (PfEMP1) and associated proteins, which are inserted in the RBC plasma membrane. The colour of a circle indicates whether or not a given gene set was enriched (dark red, *p* < 0.01; light red, 0.01 ≤ *p *< 0.05; yellow, 0.05 ≤ *p *< 0.10; green, *p* ≥ 0.10)

Thus far, the analysis has only focused on identifying alterations in the metabolism of the parasite. This section presents the results of GSEA, which includes analyses of all biological processes of the parasite. Figure 6a shows the enrichment of gene sets associated with the stress response pathways (SRPs) of the parasite. Similar gene sets were clustered together, as indicated by the grey lines showing the interactions among them. The black lines show gene sets that directly contributed to the enrichment of SRPs. To identify the metabolic changes critical for the appearance of vacuole-like structures observed during hypoxanthine deprivation, enrichment analysis was performed for transcriptomic data obtained at 24 h into the IDC. This revealed an enrichment of genes associated with *P. falciparum* RBC membrane protein 1 (PfEMP1), intracellular signalling, Maurer’s cleft, rosette formation, protein export, and mitosis.

To ascertain whether enrichment of these gene sets constitutes a general response or a response specific to hypoxanthine deprivation, GSEA was also performed on published data obtained under isoleucine deprivation at the 30-h mark, when parasites enter a hibernatory state [9]. Figure 6b shows the gene sets associated with SRPs under these conditions, with gene clusters colour-coded in a manner identical to the data obtained under hypoxanthine deprivation. As in the case of hypoxanthine deprivation, this analysis also revealed enrichment of genes associated with rosette formation, Maurer’s cleft and protein export. However, gene sets associated with PfEMP1, intracellular signalling and mitosis were not enriched as they were under hypoxanthine deprivation. A complete list of gene sets belonging to the biological process ontology of *P. falciparum*, along with their enrichment level at each time point and deprivation condition, is provided in Additional files 5 and 6.

## Discussion

### Parasite response to hypoxanthine deprivation

Parasites deprived of hypoxanthine displayed morphological and developmental aberrations during the first IDC and could not be rescued after two IDCs in the deprived medium. The formation of vacuole-like structures was evident in many parasites at 24 h into the IDC. Such vacuole formation, which has previously been observed in parasites exposed to heat stress, is attributed to autophagic programmed cell death [18]. The hypoxanthine-deprived parasites studied here also exhibited a prolonged trophozoite stage, but those that did not succumb to death ultimately established another round of IDC. This type of delayed cell cycle progression is also observed in isoleucine-deprived parasites [9]. Interestingly, parasites deprived of isoleucine are viable up to 72 h, similar to those deprived of hypoxanthine, but cannot be rescued upon transfer to an isoleucine-rich medium after four IDCs [9]. These observations suggest that *P. falciparum* is capable of effectively responding to short-term nutrient stress.

**Fig. 7:**
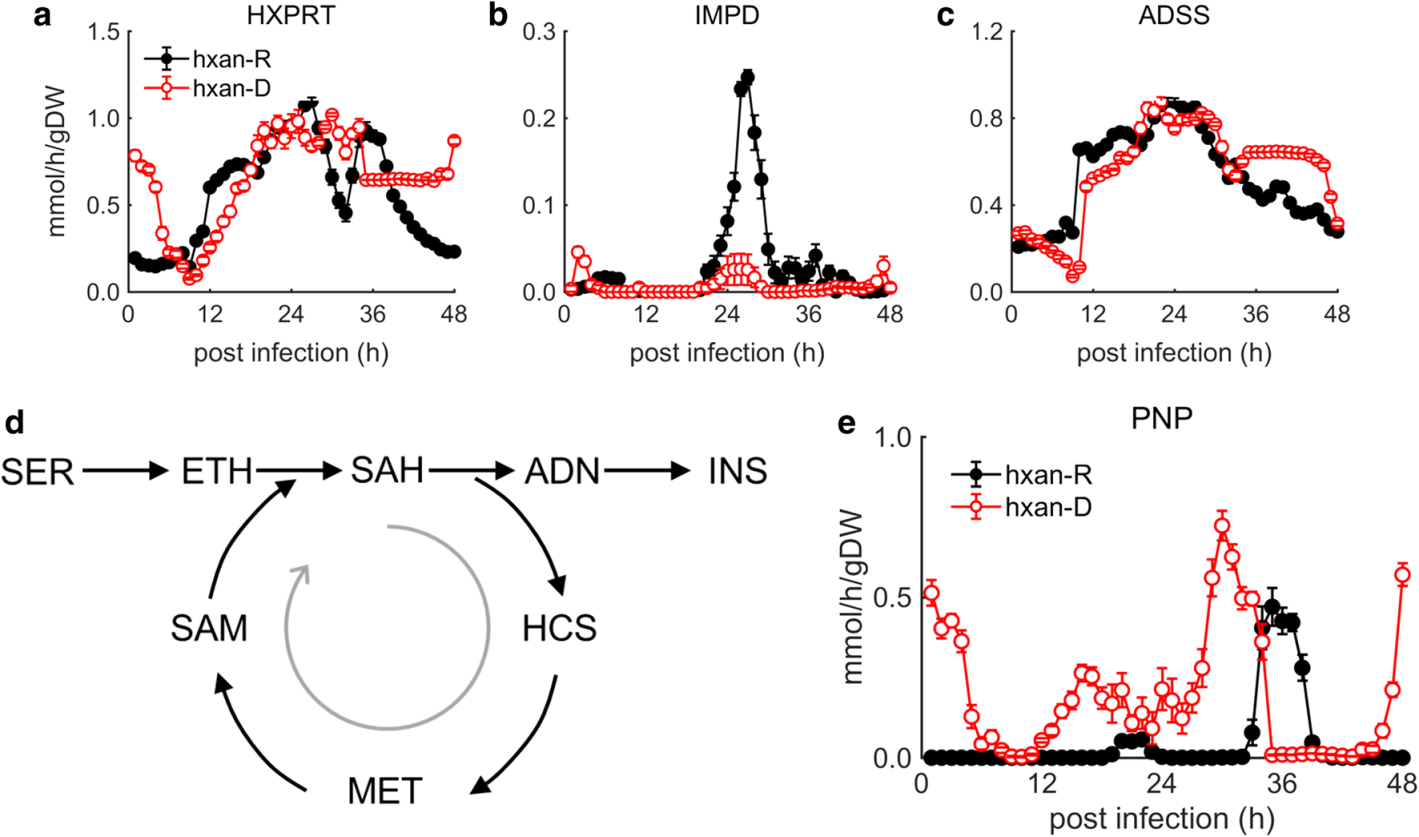
*Plasmodium falciparum* metabolic enzymes involved in the hypoxanthine salvage and recycling process. **a** Hypoxanthine phosphoribosyltransferase (HXPRT) catalyzes the formation of inosine monophosphate (IMP) from hypoxanthine. **b** IMP dehydrogenase (IMPD) catalyzes the formation of xanthine monophosphate (XMP) from IMP. **c** Adenylosuccinate synthase (ADSS) catalyzes the formation of adenylosuccinic acid from IMP. **d** Compensatory mechanism involved in maintenance of the hypoxanthine pool under deprivation conditions. **e** Purine nucleoside phosphorylase (PNP) catalyzes the formation of hypoxanthine from inosine (INS). In **a**–**c** and **e**, the ordinate shows the reaction rate of the metabolic enzyme. The error bars show the standard deviations of 50 simulations computed after adding random Gaussian noise with a mean of zero and a standard deviation of 5% to the transcriptomic data obtained under hypoxanthine-rich and hypoxanthine-deprived conditions. ADN, adenosine; ETH, ethanolamine; HCS, homocysteine; hxan-D, hypoxanthine-deprived; hxan-R, hypoxanthine-rich; MET, l-methionine; SAH, *S*-adenosylhomocysteine; SAM, *S*-adenosylmethionine; SER, l-serine

Based on model simulations, it can be inferred that the hypoxanthine-deprived parasite invokes mechanisms to maintain a sufficient pool of purine bases, which thereby supports its growth under limited availability of hypoxanthine. Model-predicted reaction fluxes that support this hypothesis are presented below (Fig. 7). First, hypoxanthine phosphoribosyltransferase (HXPRT) activity is largely unchanged during hypoxanthine deprivation (Fig. 7a). HXPRT synthesizes IMP, which is a precursor for xanthine monophosphate (XMP) and adenylosuccinic acid (ASA). Second, the parasite maintains flux through adenylosuccinate synthase (ADSS) but not through IMP dehydrogenase (IMPD) (Fig. 7c). This makes sense because the precursor for IMP is hypoxanthine, which is present in substantially less quantity in the medium (90 µM in the rich medium vs 0.5 µM in the deprived medium). Together, these results suggest that, under limited concentrations of hypoxanthine, the parasite invokes pathways that synthesize inosine, which it then uses to synthesize hypoxanthine through the PNP enzyme (Fig. 7e).

Figure 7d depicts the cycle of production of inosine through a pathway that requires cleaving of adenosine (ADN) from *S*-adenosylhomocysteine (SAH). The cycle is futile because SAH requires *S*-adenosylmethionine (SAM), which cannot be synthesized without ATP. Therefore, although the parasite can synthesize hypoxanthine through this pathway, it must also redistribute (enhance or suppress) reactions associated with ATP to maintain the synthesis of purine-based nucleic acids. The deprived parasites suppressed metabolic fluxes through ATP-consuming reactions, such as those of phosphoenolpyruvate carboxykinase (PPCK), adenylate kinase and carbamoyl-phosphate synthase, while also increasing metabolic fluxes through certain ATP-consuming reactions, such as those of glutathione synthase and guanylate kinase (Additional file 1: Fig. S2).

Interestingly, the parasites can also produce hypoxanthine through methylthioadenosine (Fig. 1), which is produced during polyamine synthesis. However, there was no increase in metabolic flux through this pathway under the hypoxanthine-deprived condition. In fact, hypoxanthine-deprived parasites produced significantly less putrescine, which is captured in Fig. 5e. The strategy behind producing hypoxanthine via inosine instead of methyladenosine is not clear.

### Model-predicted consequences of the compensatory response

**Fig. 8:**
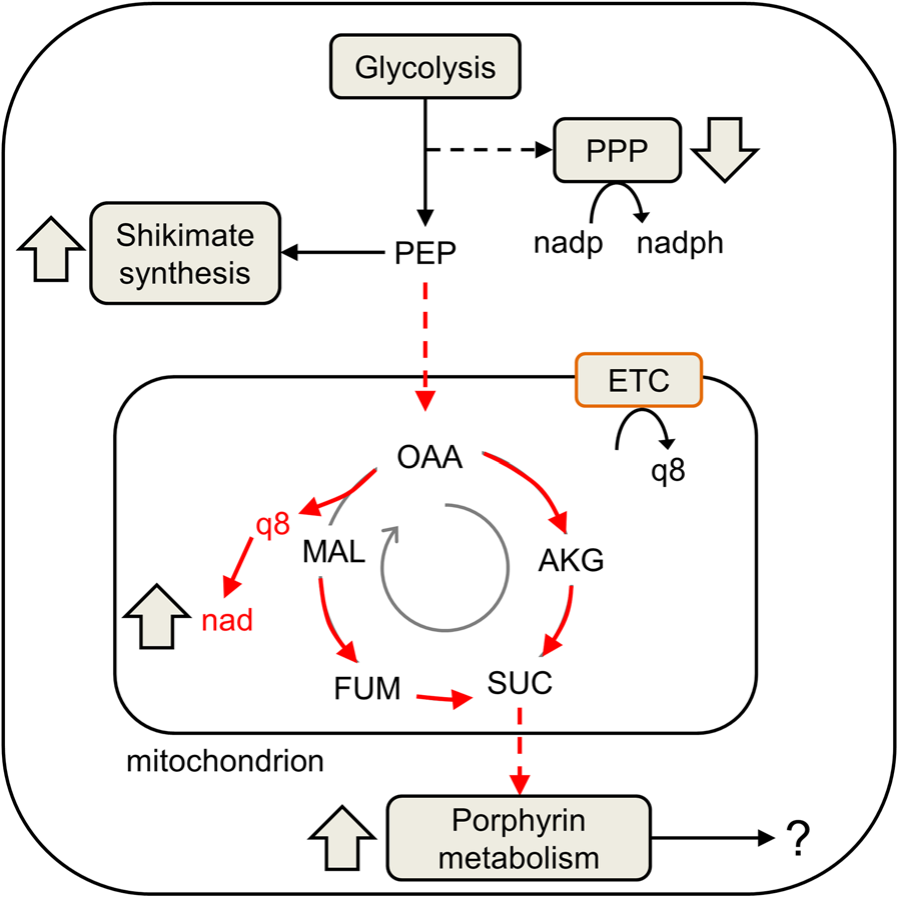
Consequences of hypoxanthine deprivation on parasite metabolism, as captured in model simulations. These are most clearly delineated in model simulations of parasite metabolism during the first 24 h of hypoxanthine deprivation. The simulations indicated that the parasite supplies phosphoenolpyruvate (PEP) to synthesize chorismate, which is a precursor for ubiquinone synthesis as part of shikimate synthesis. PEP is also utilized to synthesize oxaloacetate (OAA), which in turn leads to enhanced porphyrin metabolism. The red arrows indicate the metabolic flow occurring under hypoxanthine-deprivation conditions. A dotted arrow indicates more than one reaction step. AKG, alpha-ketoglutarate; ETC, electron transport chain; FUM, fumarate; MAL, l-malate; nad, oxidized nicotinamide adenine dinucleotide; nadp, oxidized nicotinamide adenine dinucleotide phosphate; nadph, reduced nadp; PPP, pentose phosphate pathway; SUC, succinate; q8, ubiquinone

The futile cycle discussed above requires SAM, which is a co-substrate for enzymes (i.e., 3-demethylubiquinone-9, 3-*O*-methyltransferase and ubiquinone/menaquinone biosynthesis C-methyltransferase) and is essential for ubiquinone synthesis. Therefore, an increase in flux through SAM should also increase flux through reactions associated with ubiquinone synthesis and create a demand for the precursors necessary for ubiquinone synthesis, such as chorismate. One of the top pathways significantly altered under hypoxanthine deprivation in the simulations was that of shikimate biosynthesis (Table 2), of which chorismate is a by-product. Therefore, chorismate is also expected to increase during hypoxanthine deprivation (Fig. 8).

An increase in shikimate synthesis should also create a demand for its precursor, phosphoenolpyruvate (PEP), and, consequently, influence other reactions that require PEP. PEP is also an important precursor in the methylerythritol phosphate pathway (MEP) pathway of isoprenoid synthesis, and as with shikimate synthesis, the flux through the MEP pathway significantly increased under hypoxanthine deprived conditions (Table 2). Interestingly, the flux through PPCK (Additional file 1: Fig. S2), an ATP-consuming reaction that converts oxaloacetate (OAA) back to PEP, decreased, suggesting a buildup of OAA.

Figure 8 shows that all fluxes downstream of cytosolic OAA (red arrows in the mitochondrion; e.g., malate:quinone oxidoreductase, fumarate hydratase, etc.) increased under hypoxanthine-deprivation conditions. In fact, in simulations of hypoxanthine-deprived parasites, the tricarboxylic acid (TCA) cycle seemed to deviate from the cyclic mode; specifically, malate:quinone oxidoreductase and fumarate hydratase worked in the reverse direction under deprived conditions (Fig. 8, red arrows). This deviation suggests an increase in oxidized nicotinamide adenine dinucleotide (nad). The metabolic flux through the oxidative branch of the pentose phosphate pathway (PPP) also decreased significantly under hypoxanthine-deprivation conditions. This suggests that the increased haem biosynthesis, together with a reduction in PPP, causes an unfavourable increase in oxidative stress of the parasite under deprivation conditions. Notably, metabolic reactions associated with haem degradation and minimization of oxidized glutathione (glutathione reductase and glutathione efflux) also increased, suggesting an increase in oxidative stress under deprivation conditions (Additional file 1: Fig. S3).

## Conclusion

*Plasmodium falciparum* lacks the machinery to synthesize purine bases de novo and relies on purine salvage to meet its need [19]. The present study showed that, under purine-deprivation conditions, the malaria parasite *P. falciparum* utilizes pathways to recycle available purine and maintain DNA replication. However, enhanced flux through the pathways that recycle purines causes an imbalance in redox and mitochondrial metabolism, which is detrimental to the parasite. These findings suggest the following conclusions:

1. Maintenance of redox reactions and mitochondrial function within their normal range is essential for *P. falciparum* survival during the blood stage;
2. *Plasmodium falciparum* redistributes metabolic reactions to maintain synthesis of purine-based nucleic acids under limited concentrations of hypoxanthine;
3. The compensatory mechanisms, induced under hypoxanthine-deprivation conditions, cause irreparable damage to *P. falciparum* by making them unviable for replication after 72 h of deprivation, despite supplementation with a hypoxanthine-rich medium.

## Additional files

**Additional file 1.** Supplementary computational details Text S1–S2 and additional Figures S1–S3.

**Additional file 2.** Significantly altered erythrocyte reactions in response to parasite infection and hypoxanthine concentration.

**Additional file 3.** List of metabolites detected under hypoxanthine-rich and -deprived conditions.

**Additional file 4.** Significantly altered metabolic genes and reactions of *Plasmodium falciparum* in response to hypoxanthine deprivation.

**Additional file 5.** Gene set enrichment analysis of transcriptomic data from hypoxanthine-deprived *Plasmodium falciparum.*

**Additional file 6.** Gene set enrichment analysis of transcriptomic data from isoleucine-deprived *Plasmodium falciparum.*

## Abbreviations

ADN: adenosine
ADP: adenosine diphosphate
ADSS: adenylosuccinate synthase
AKG: alpha-ketoglutarate
AMP: adenosine monophosphate
ASA: adenylosuccinic acid
ATP: adenosine triphosphate
CoA: coenzyme A
ET: early trophozoite
ETC: electron transport chain
ETH: ethanolamine
FV: food vacuole
GMP: guanosine monophosphate
GSEA: gene set enrichment analysis
HCS: homocysteine
HCT: haematocrit
hxan-D: hypoxanthine-deprived
hxan-R: hypoxanthine-rich
HXPRT: hypoxanthine phosphoribosyltransferase
IDC: intra-erythrocytic developmental cycle
IMP: inosine monophosphate
IMPD: IMP dehydrogenase
LT: late trophozoite
MACS: magnetic activated cell sorting
MAL: l-malate
MEP: methylerythritol phosphate pathway
MET: l-methionine
nad: oxidized nicotinamide adenine dinucleotide
nadp: oxidized nicotinamide adenine dinucleotide phosphate
nadph: reduced nadp
OAA: oxaloacetate
PEP: phosphoenolpyruvate
PfEMP1: *P. falciparum* RBC membrane protein 1
PNP: purine nucleoside phosphorylase
PPC: phosphoenolpyruvate carboxylase
PPCK: phosphoenolpyruvate carboxykinase
PPP: pentose phosphate pathway
q8: ubiquinone
R: ring
RBC: red blood cell
RPMI: Roswell Park Memorial Institute
S: schizont
SAH: *S*-adenosylhomocysteine
SAM: *S*-adenosylmethionine
SER: l-serine
SRP: stress response pathway
SUC: succinate
TCA: tricarboxylic acid
UPLC: ultrahigh-performance liquid chromatography
XMP: xanthine monophosphate
