# Supplementary material for: Short-term metabolic adjustments in Plasmodium falciparum counter hypoxanthine deprivation at the expense of long-term viability
Source: Malar J. 2019 Mar 19;18:86. doi: 10.1186/s12936-019-2720-3 (PMC6423861; doi:10.1186/s12936-019-2720-3)
Supplement: Supplementary file 1 — Additional file 1. Supplementary computational details Text S1–S2 and additional Figures S1–S3. [file 12936_2019_2720_MOESM1_ESM.docx]

**Additional file 1**

Table of Contents

**Text S1. Using metabolomic data to estimate RBC reaction fluxes**

**Text S2. Metabolomic data extraction and compound identification**

**Figure S1. RBC re-infection in response to different hypoxanthine concentrations and deprivation times**

**Figure S2. ATP-consuming reactions of *P. falciparum* under hypoxanthine-rich and -deprived conditions**

**Figure S3. Metabolic reactions involved in oxidative stress of *P. falciparum***

**References**

# Text S1. Using metabolomic data to estimate RBC reaction fluxes

The present study used a recently described method [1] that incorporates metabolomic data from uninfected and infected RBCs to predict condition-specific RBC metabolism. Briefly, it was assumed that *1*) flux through an enzyme is proportional to the amount of substrate participating in the enzymatic reaction and *2*) a substrate with the lowest concentration is rate limiting for a reaction with multiple substrates. If a reaction is unidirectional, then these assumptions are true for the products as well.

To implement these assumptions and make use of the available metabolomic data, the level of each metabolite participating in a given reaction was represented relative to its median value taken over the time course of the experiment, i.e., 48 h. Second, the minimum of the relative values of metabolites participating in a metabolic reaction was obtained to modify the reaction flux at a given time point of the intraerythrocytic developmental cycle (IDC). Mathematically, the following equations were solved:

$$\begin{aligned} \delta^{t}=\min\left[ \sum_{i\in R} v_{i}^{t}- \alpha_{i}^{t}\cdot v_{i,nom}+ \sum_{j\in N} v_{j}^{t}-v_{j,nut}^{t}] \right] \#\left( 1 \right) \end{aligned}$$

subject to: $\text{S}\cdot\text{v}=0$ and $\text{v}_{lb}\leq\text{v}\leq\text{v}_{ub}$

$$\begin{aligned} \min\sum_{i\in M} \left| v_{i}^{t}-v_{i,nom} \right| \#\left( 2 \right) \end{aligned}$$

subject to: $\sum_{i\in R} v_{i}^{t}- \alpha_{i}^{t}\cdot v_{i,nom}+ \sum_{j\in N} v_{j}^{t}-v_{j,nut}^{t}\leq\delta^{t}$

$\text{S}\cdot\text{v}=0$ and $\text{v}_{lb}\leq\text{v}\leq\text{v}_{ub}$

Here, $\text{S}$ is a matrix containing the stoichiometry of red blood cell (RBC) metabolic reactions. $\alpha^{t}$ is a column vector with each row-element containing the minimal value of all the metabolites taking part in the row reaction at a given time *t*; before taking the minimal value of the participating metabolites, each metabolite is normalized by its median value during the IDC. *R* denotes a set of unidirectional reactions, *N* denotes the set of reactions associated with nutrient uptake/secretion, and *M* represents the set of all metabolic reactions in the RBC. $\text{v}_{lb}$and $\text{v}_{ub}$ are vectors representing the lower and upper bounds, respectively, of metabolic reactions in the RBC. $v_{i,nom}$ and $v_{j,nom}$ denote the *i*^th^ and *j*^th^ values, respectively, of the column vector ($v_{nom}$) containing the nominal values of metabolic reactions in the RBC. $v_{j,nut}^{t}$ represents the *j*^th^ value of the column vector ($v_{nut}^{t}$) containing the optimal rates of nutrient uptake/secretion by the erythrocyte at a given time *t*. The reader is referred to a previous report [1] for details of the optimization method used to compute $v_{nom}$ and $v_{nut}^{t}$.

# Text S2. Metabolomic data extraction and compound identification

Raw data were extracted, peaks were identified, and quality check (QC) was performed using Metabolon’s hardware and software. Compounds were identified by comparison to Metabolon’s library entries of purified standards or recurrent unknown entities. Biochemical identification was based on three criteria: a retention index (RI) within a narrow RI window of the proposed identification, accurate mass match to the library +/– 10 ppm, and the MS/MS forward and reverse scores between the experimental data and authentic standards. The MS/MS score was based on a comparison of the ions present in the experimental spectrum to those present in the library spectrum. Although a molecule may resemble another based on one of these factors, the use of all three factors can distinguish and differentiate biochemicals.

# Figure S1. RBC re-infection in response to different hypoxanthine concentrations and deprivation times

The present study investigated the ability of parasites to re-infect RBCs after hypoxanthine deprivation during the first IDC. The parasites were cultured at a hypoxanthine concentration of 0, 0.5, 2, 5 or 90 µM. At 40 hrs, a tenth of each culture was transferred to hypoxanthine-rich medium (90 µM). Rings and young trophozoites were seen at 72 hrs, indicating that parasites cultured at or below 5 µM of hypoxanthine were able to establish a second IDC in hypoxanthine-rich medium. Surprisingly, parasites cultured without hypoxanthine during the first 40 hrs were able to re-infect RBCs upon transfer to hypoxanthine-rich medium, albeit at lower numbers. Overall, a decrease in hypoxanthine concentration during the first IDC caused a corresponding decrease in the number of parasites during the second IDC.

**
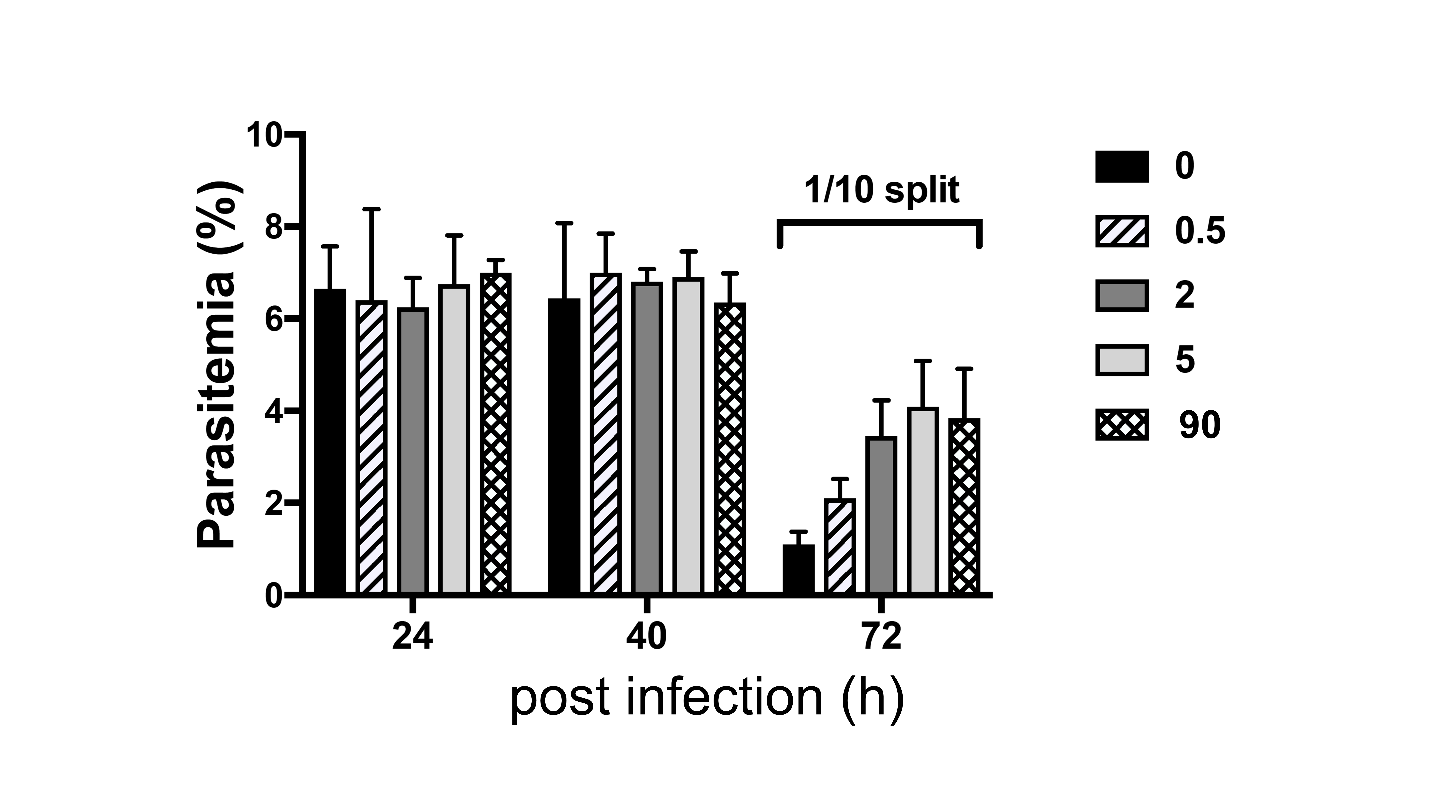
**

# Figure S2. ATP-consuming reactions of *P. falciparum* under hypoxanthine-rich and -deprived conditions

A mechanism that allows the hypoxanthine-deprived parasites to maintain synthesis of purine-based nucleotides was presented in the main text. However, this mechanism, which involves the use of adenosine triphosphate (ATP), implies no *de novo* synthesis of ATP but only a re-distribution of available ATP to maintain the synthesis of purine-based nucleotides. Figure S3 shows some important ATP-consuming reactions of *P. falciparum* that were altered under hypoxanthine-deprived conditions. Phosphoenolpyruvate carboxykinase (PPCK) synthesizes phosphoenolpyruvate from oxaloacetate, carbamoyl-phosphate synthase (CBPS) synthesizes carbamoyl-phosphate, and glutathione synthase (GTHS) synthesizes glutathione, each using ATP. These simulations suggest that the parasite was slowing down processes that required ATP and only focused on processes that were important for survival, e.g., GTHS, which is important for scavenging free radicals [2].

**
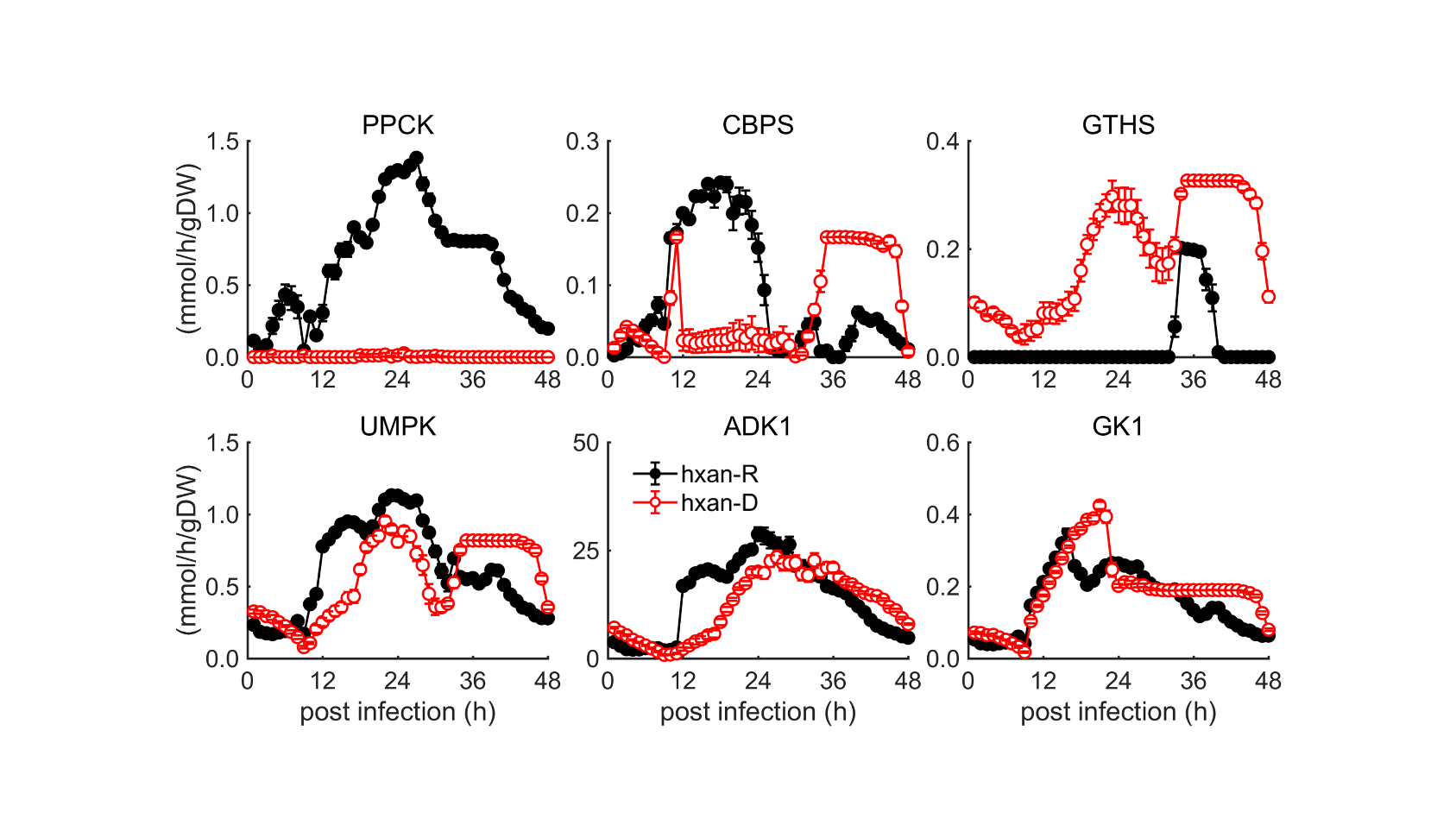
**

# Figure S3. Metabolic reactions involved in oxidative stress of *P. falciparum*

As discussed above, the parasite synthesized more glutathione under hypoxanthine-deprived conditions. There was also an increase in haem degradation (HEMED), which detoxifies haem utilizing (reduced) glutathione, suggesting an increase in oxidative stress under deprivation conditions. The glutathione-disulfide reductase (GTHOr) reaction rate increased, presumably to maintain the availability of reduced glutathione for HEMED activity [3]. It is suggested that hypoxanthine-deprived parasites suffer from increased oxidative stress, because in addition to an increase in GTHS, HEMED, and GTHOr, the efflux of oxidized glutathione from the parasite cytosol (GTHOXti) also increases. Energy-depleted parasites have been shown to export oxidized glutathione from their cytosol [4].

**
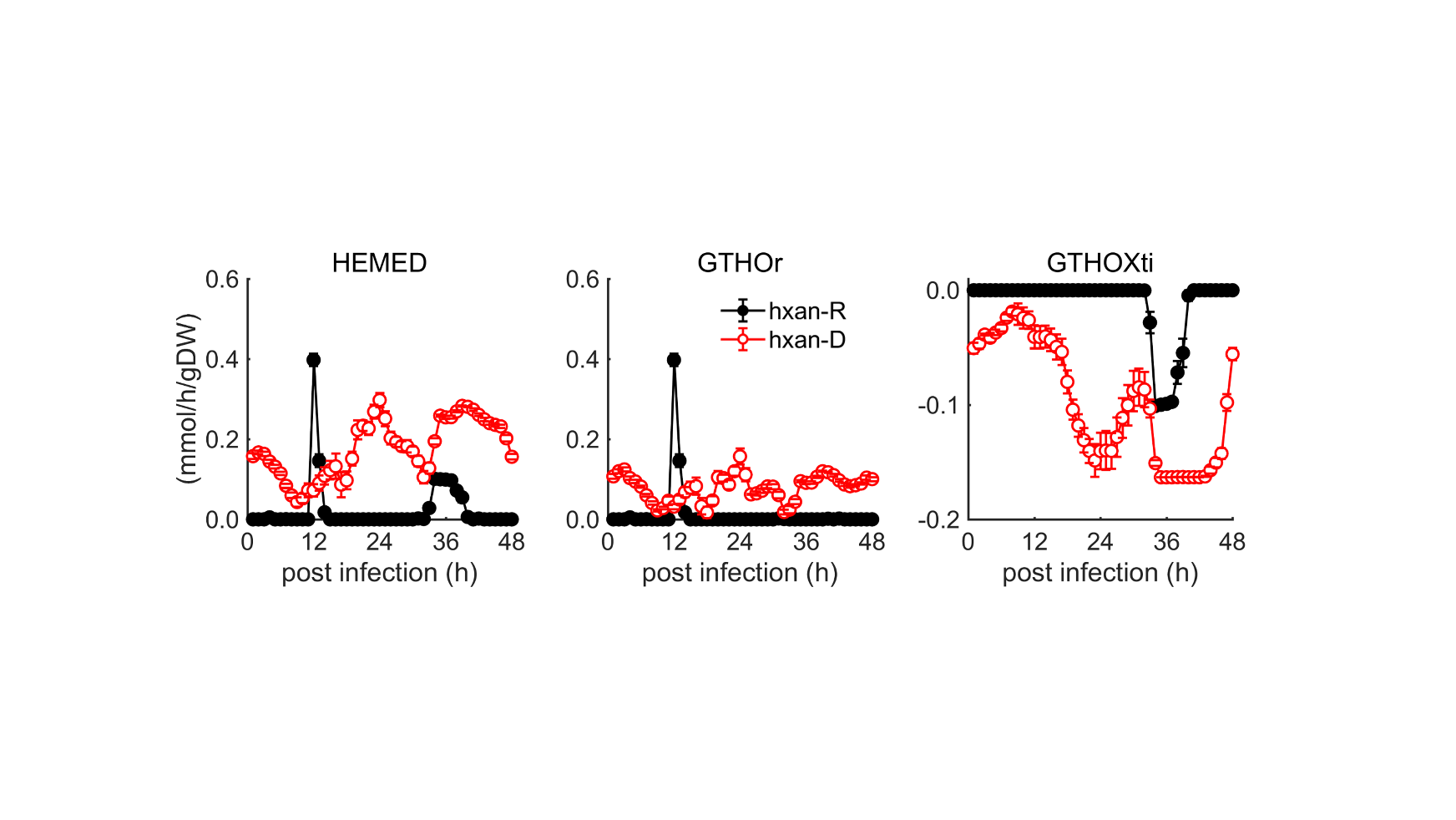
**

# References

1. Tewari SG, Swift R, Oyama T, Rajaram K, Reifman J, Prigge ST, Wallqvist A: **The fumarate recycling in *Plasmodium falciparum* promotes maintenance of redox metabolism during the asexual cycle.** Under review.

2. Muller S: **Role and regulation of glutathione metabolism in *Plasmodium falciparum*.** *Molecules* 2015, **20:**10511-10534.

3. Atamna H, Ginsburg H: **Heme degradation in the presence of glutathione. A proposed mechanism to account for the high levels of non-heme iron found in the membranes of hemoglobinopathic red blood cells.** *J Biol Chem* 1995, **270:**24876-24883.

4. Barrand MA, Winterberg M, Ng F, Nguyen M, Kirk K, Hladky SB: **Glutathione export from human erythrocytes and *Plasmodium falciparum* malaria parasites.** *Biochem J* 2012, **448:**389-400.
